# Potential Effects of CXCL9 and CCL20 on Cardiac Fibrosis in Patients with Myocardial Infarction and Isoproterenol-Treated Rats

**DOI:** 10.3390/jcm8050659

**Published:** 2019-05-11

**Authors:** Chao-Feng Lin, Chih-Jou Su, Jia-Hong Liu, Shui-Tien Chen, Han-Li Huang, Shiow-Lin Pan

**Affiliations:** 1 Ph.D. Program for Cancer Molecular Biology and Drug Discovery, College of Medical Science and Technology, Taipei Medical University and Academia Sinica, Taipei 110, Taiwan; thcpci@gmail.com (C.-F.L.); jojocat127@gmail.com (C.-J.S.); 2 Department of Medicine, MacKay Medical College, New Taipei City 252, Taiwan; 3 Division of Cardiology, Department of Internal Medicine, MacKay Memorial Hospital, Taipei 104, Taiwan; 4 Graduate Institute of Cancer Molecular Biology and Drug Discovery, College of Medical Science and Technology, Taipei Medical University, Taipei 110, Taiwan; horng428@gmail.com; 5 Institute of Biological Chemistry, Academia Sinica, Taipei 115, Taiwan; bcchen@gate.sinica.edu.tw; 6 TMU Biomedical Commercialization Center, Taipei Medical University, Taipei 110, Taiwan; 7 Ph.D. Program in Biotechnology Research and Development, College of Pharmacy, Taipei Medical University, Taipei 110, Taiwan

**Keywords:** myocardial infarction, cardiac fibrosis, CXCL9, CCL20, isoproterenol

## Abstract

The chemokines CXCL9 and CCL20 have been reported to be associated with ventricular dysfunction. This study was aimed to investigate the effects of CXCL9/CCL20 on cardiac fibrosis following myocardial infarction (MI). Blood samples of patients with MI were obtained to determine the serum CXCL9, CCL20, tumor necrosis factor-α (TNF-α), and transforming growth factor-β (TGF-β). The expression of CXCL9 and CCL20 in hypoxia-incubated H9c2 cells and TNF-α/TGF-β-activated peripheral blood mononuclear cells (PBMCs) were examined. The experimental MI of rats was produced by the intraperitoneal injection of isoproterenol (ISO) (85 mg/kg/day) for two consecutive days. The growth and migration of CXCL9/CCL20-incubated cardiac fibroblasts in vitro were evaluated. TNF-α/TGF-β-activated PBMCs showed an enhanced expression of CXCL9 and CCL20, while hypoxic H9c2 cells did not. Patients with MI had significantly enhanced levels of serum TGF-β and CXCL9 compared to healthy subjects. ISO-treated rats had increased serum CXCL9 levels and marked cardiac fibrosis compared to control rats. The trend of increased serum CCL20 in patients with MI and ISO-treated rats was not significant. CXCL9-incubated cardiac fibroblasts showed enhanced proliferation and migration. The findings of this study suggest that an enhanced expression of CXCL9 following MI might play a role in post-MI cardiac fibrosis by activating cardiac fibroblasts.

## 1. Introduction 

Myocardial infarction (MI) is the leading cause of death in the world. It is mainly caused by coronary artery occlusion that results in dysfunctional myocardium [1,2]. Despite restoration of coronary circulation, ischemic cell death eventually leads to the formation of post-MI cardiac fibrosis in which the damaged myocardial tissue is replaced with fibrotic scar [3,4]. Post-MI cardiac fibrosis is a complex process of remodeling in the myocardium and has been identified as an important risk factor of ventricular dysfunction, heart failure (HF), and poor prognosis [3,4]. Therefore, the prevention of post-MI cardiac fibrosis is a potential target of pharmacological treatment to maintain ventricular function and to improve clinical outcomes following MI.

Various cytokines and chemokines are involved in the process of post-MI cardiac fibrosis. Tumor necrosis factor-α (TNF-α) and transforming growth factor-β (TGF-β) are key cytokines that increase rapidly following MI and mediate activation of chemokine cascades [3,5,6]. Chemokines are a family of small cytokines and can be divided into four groups: CXC, CC, C, and CX3C. These groups are characterized by the number of amino acids located between the N-terminal cysteine residues [7]. The enhanced expression of TNF-α, TGF-β, and chemokines following MI may mediate the recruitment of leukocytes into damaged myocardium, resulting in the clearance of dead cardiomyocytes and extracellular debris, as well as the modulation of the repopulation of immune cells and fibroblasts [3,5,6,8,9,10,11,12]. All these events contribute to fibrotic changes of the myocardium and ventricular dysfunction.

Several previous studies have reported that some specific chemokines (i.e., CXCL9 and CCL20) were related to ventricular dysfunction and the severity of ischemic heart disease (IHD) [13,14,15,16,17,18,19]. CXCL9 was associated with progression of atherosclerosis [15,16] and some types of cardiomyopathy [17,18]. CCL20 has been reported to be a biomarker in patients with IHD [19]. Despite these findings, the roles of CXCL9 and CCL20 in the pathogenesis of post-MI cardiac fibrosis remain unclear. The present study was designed to investigate the potential effects of CXCL9 and CCL20 on cardiac fibrosis following MI by using an animal model of isoproterenol (ISO)-induced MI and analyzing blood samples of patients with MI.

## 2. Materials and Methods

### 2.1. Cell Culture and Reagents

H9c2 cells were obtained from Bioresource Collection and Research Center (Hsinchu, Taiwan). Human peripheral blood mononuclear cells (PBMCs) were obtained from healthy volunteers of our laboratory. Normal human cardiac fibroblasts (NHCFs) were obtained from Lonza Group (Walkersville, MD, USA). Protein array kits were purchased from R & D System (Minneapolis, MN, USA). Isoproterenol (ISO) and sulforhodamine B (SRB) were purchased from Sigma-Aldrich, Inc. (St. Louis, MO, USA). Hematoxylin and Eosin (HE) staining and trichrome staining were performed under the help of Rapid Science Co., Ltd. (Taichung, Taiwan). Recombinant human TNF-α, TGF-β, CXCL9, and CCL20 were purchased from Peprotech (Rocky Hill, NJ, USA).

### 2.2. Blood Samples of Patients with MI

This study was approved by the institutional review board of MacKay Memorial Hospital (Taipei, Taiwan; approved no. 16MMHIS113e, 17MMHIS074e). Patients who admitted for the primary diagnosis of acute MI were enrolled. We excluded the patients who had a history of HF, cancer, concomitant infection, and previous MI. Patients who had previous history of coronary artery disease receiving percutaneous coronary intervention (PCI) or surgical revascularization were also excluded. Each patient with MI received standard of care, including successful PCI to restore coronary circulation of infarct-related artery (achievement of Thrombolysis in Myocardial Infarction (TIMI) grade flow 3), antiplatelet agents, and heparin therapy. Blood samples were obtained before PCI (pre-PCI) and 3 h after coronary reperfusion (post-PCI) for enzyme-linked immunosorbent assays (ELISA) to determine the serum levels of biomarkers, including CXCL9, CCL20, TNF-α, and TGF-β. Additionally, blood samples of healthy volunteers were analyzed for comparison. Informed consent were obtained from all healthy volunteers and patients. All subjects gave their informed consent before they participated in the study.

### 2.3. Protein Array

Blood samples of 2 patients with MI were selected randomly and used for an initial screen to evaluate the expression of CXCL9 and CCL20 among various proteins provided by protein array chip. Briefly, samples were exposed to a cocktail of biotinylated antibodies and then incubated with the array membrane. Captured proteins were visualized using chemiluminescent detection reagents and were quantified using ImageJ software to calculate the altered fold. The results were evaluated according to the duration of exposure to detection antibodies (short and long exposure). The altered fold was calculated based on the data of long exposure.

### 2.4. ELISA

ELISA was used to detect the expression of CXCL9 (#ELH-MIG, RayBiotech, Peachtree Corners, GA, USA), CCL20 (#ELH-MIP3a, RayBiotech), TNF-α (#ELH-TNFa-1, RayBiotech), and TGF-β (#ELH-TGFb1-1, RayBiotech) in human plasma or culture medium and used to detect the expression of N-terminal prohormone of brain natriuretic peptide (NT-proBNP) (#HEA485Ra, Cloud-Clone, Katy, TX, USA), CXCL9 (#MBS452489, MyBioSource, San Diego, CA, USA), and CCL20 (#MBS175952, MyBioSource) in rat plasma. Briefly, samples were collected and put into anti-coagulant containing tube. After centrifuging, the aliquot supernatant was collected and 100 µL was added into the wells for incubation. After the reaction, wells were washed by wash buffer and then treated sequentially by adding detection antibody. Finally, tetramethylbenzidine (TMB) substrate was used for color production. The results were measured by spectrophotometer at wavelength 450 nm.

### 2.5. TNF-α- and TGF-β-Activated PBMCs

PBMCs were incubated in a Roswell Park Memorial Institute (RPMI) medium supplemented with 10% fetal bovine serum (FBS). After incubating with different concentrations (1, 10, 100 pg/mL) of recombinant human TNF-α and TGF-β, the expression of CXCL9 and CCL20 in a medium was determined by ELISA.

### 2.6. Hypoxia-Incubated H9c2 Cells

H9c2 cells were found to be similar to normal primary cardiomyocytes with regard to their energy metabolism features and were commonly used as the in vitro model of cardiac hypoxia and simulated MI [20]. In the present study, H9c2 cells were cultured in a medium supplemented with 10% FBS at 37 °C in a 95% air/5% CO_2_ atmosphere in culture flasks. The medium was freshly replaced every 3 days and was supplemented with streptomycin (100 mg/mL) and penicillin (100 IU/mL). A simulated hypoxic stress was produced by exposure to 5% CO_2_ and 95% N_2_ in a closed chamber for 24 h. The culture medium was collected to evaluate the expression of CXCL9 and CCL20 by ELISA.

### 2.7. The Growth and Migration of NHCFs Stimulated by CXCL9 and CCL20

NHCFs were incubated in a 10% fibroblast basal medium supplemented with GA-1000, insulin 0.5%, rhFGF-B, and 10% FBS. After incubating with different concentrations (1, 10, and 100 ng/mL) of recombinant human CXCL9 and CCL20 for 72 or 96 h, the cell proliferation of NHCFs were examined by using an SRB assay. The SRB assay relies on the property of SRB, which binds stoichiometrically to cellular proteins under mild acidic conditions and then can be extracted using basic conditions; thus, the amount of bound dye can be used as a proxy for cell mass and proliferation [21]. In brief, after incubation with vehicle or test compounds for the indicated time interval, cells were fixed with 10% TCA and stained with SRB at 0.4% (w/v) in 1% acetic acid. Excess SRB was washed away by 1% acetic acid, and stained cells were lysed with a 10mM Trizma base. The absorbance was measured at a wavelength of 515 nm.

In the migration assay, 5 × 10^4^ NHCF cells were firstly seeded in a 12-well plate. After cell adhesion, NHCF cells were stimulated with a 10 ng/ml CXCL9 and/or CCL20 in a 1% FBS basal medium for 24 h. Cells were then trypsinized and seeded (1 × 10^4^/well) into upper chamber 6.5 mm transwell with an 8.0 µm pore polycarbonate membrane insert in a 24-well format (Corning Inc.; Corning, NY, USA) and allowed to migrate for 24 h. Membranes then fixed with 10% neutral buffered formalin on ice for 8 min and stained with crystal violet (Sigma-Aldrich; St. Louis, MO, USA) at room temperature for 20 min. Membranes were washed with water twice, and no-migrated cells were scrap off by cotton stick. Crystal violet of migrated cells was photographed and dissolved using 33% acetic acid and then quantified by detecting the absorbance at wavelength 600 nm.

### 2.8. ISO-Induced MI and Post-MI Cardiac Fibrosis in Rats

All animal experimental procedures were conducted under protocols approved by the Institutional Animal Care and Use Committee of Taipei Medical University (IACUC approved No. LAC-2017-0167). Adult male Sprague-Dawley rats (weighing 220–240 g) were obtained from Laboratory Animal Center of Taipei Medical University and were cared with constant temperature and a 12-hour light cycle. ISO-induced MI and subsequent cardiac fibrosis is a well-developed, non-surgical animal model in rats [22,23,24]. Briefly, rats were randomly assigned to the treatment group and control group. Rats in the treatment group were injected intraperitoneally with ISO (85 mg/kg body weight every 24 h for consecutive two days; Day 1 and 2), while rats in the control group were injected intraperitoneally with 0.9% normal saline. All rats were euthanized on the 14^th^ day (Day 14) after the first dose of ISO. Blood samples were obtained to determine the serum levels of CXCL9, CCL20, and NT-proBNP by ELISA. The principles and methods of ELISA to determine the serum levels of CXCL9, CCL20, and NT-proBNP in rats were similar to that of human except specific antibodies.

### 2.9. Histology (HE Stain, Trichrome Stain, and Quantification of Fibrotic Area)

HE staining was used to observe the morphology of myofibrils in the excised heart. The heart sections were rehydrated in hematoxylin solution for 20–40 min. After washing in tape water, 70% ethanol containing 1% HCl was used to remove excess dye, allowing nuclear details to emerge. Then the specimens were stained in eosin solution for 10 minutes.

Trichrome stain was used to evaluate the extent of myocardial fibrosis. Briefly, the heart sections were processed with an iron hematoxylin solution, a scarlet-acid fuchsin solution, and a phosphomolybdic-phosphotungstic acid solution in turn provided by manufactory kits. Then, the specimens were transferred directly to aniline blue solution and differentiated in 1% acetic acid solution for 5 minutes. The fibrotic area of hearts, defined as quantification of trichrome statin, was determined by the average stained pixels^2^ per total pixels^2^ in images of the heart sections (calculated by TissueFAX software; 5 images used per animal).

### 2.10. Statistics

The serum levels of CXCL9, CCL20, TNF-α, and TGF-β in patients with MI were compared to that of healthy controls (volunteers in our laboratory). Additionally, the serum levels of CXCL9, CCL20, TNF-α, and TGF-β in post-PCI status were also examined and compared to that of pre-PCI status. The expression of CXCL9 and CCL20 in hypoxia-incubated H9c2 cells and normoxia-incubated H9c2 cells were recorded and compared. Growth and proliferation of NHCFs incubated by CXCL9 and CCL20 were compared to that incubated by vehicle. The serum expression of CXCL9, CCL20, and NT-proBNP of ISO-treated rats were compared to that of control rats. After trichrome staining, the fibrotic areas in excised hearts of ISO-treated rats were compared to that of control rats. Categorical data are presented as mean ± SEM or as percentage of basal. Statistical comparisons between groups were performed using the student’s t-test and one-way ANOVA. *p* < 0.05 was considered statistically significant (* *p* < 0.05, ** *p* < 0.01, *** *p* < 0.001, **** *p* < 0.0001).

## 3. Results

### 3.1. Determination of CXCL9 and CCL20 in Patients with MI

**Figure 1:**
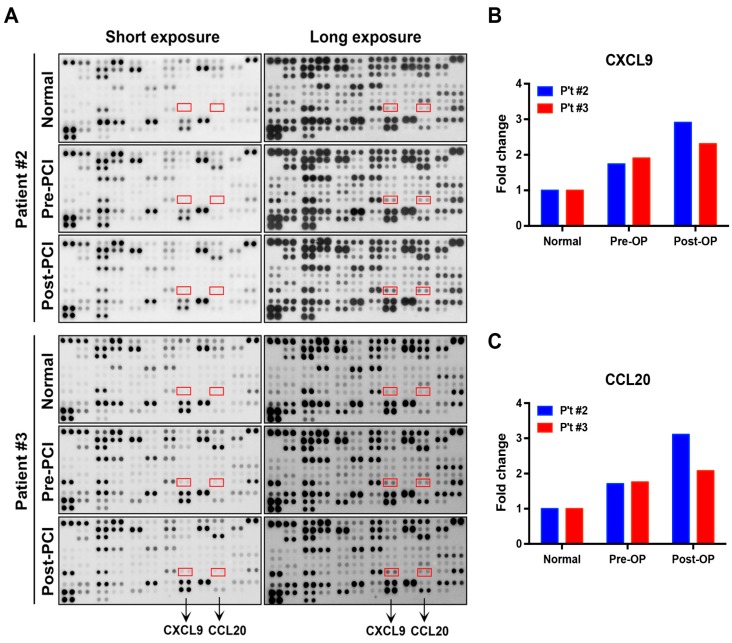
Protein array analysis evaluating the expression of CXCL9 and CCL20 among various detected protein in patients with MI (patient #2 and #3) and normal subjects (healthy volunteers in our laboratory). (**A**) The expression of CXCL9 and CCL20 consistently increased in samples of short- and long-term exposures. (**B**) Fold change of CXCL9 expression in patient #2 and #3. (**C**) Fold change of CCL20 expression in patient #2 and #3. MI = myocardial infarction; PCI = percutaneous coronary intervention; Post-PCI = 3 h after coronary reperfusion by PCI; Pre-PCI = before PCI.

Blood samples of two patients with MI (patients #2 and #3) were selected randomly and analyzed by a protein array to detect the expression of chemokines (Figure 1A). Among the various detected proteins provided by the manufacturer’s chip, the results from both the short and long exposures consistently showed an enhanced expression of CXCL9 and CCL20 in patients with MI (Figure 1A).

The quantification of the protein array results showed that both MI patients had an elevated serum level of CXCL9 and CCL20 compared to normal individuals. Additionally, the expression of serum CXCL9 and CCL20 following PCI were greater than that pre-PCI (Figure 1B,C). Compared to normal individuals, patient #2 had a 1.74-fold increase in pre-PCI CXCL9 and a 2.91-fold increase in post-PCI CXCL9 (Figure 1B); patient #3 had a 1.91-fold increase in pre-PCI CXCL9 and a 2.31-fold increase in post-PCI CXCL9 (Figure 1B). Compared to normal individuals, patient #2 had a 1.71-fold increase in pre-PCI CCL20 and a 3.11-fold increase in post-PCI CCL20 (Figure 1C); patient #3 had a 1.76-fold increase in pre-PCI CCL20 and a 2.08-fold increase in post-PCI CCL20 (Figure 1C). Based on this initial analysis by protein array, we determined CXCL9 and CCL20 as the target chemokines for the following study.

### 3.2. CXCL9, CCL20, and TGF-β Remained Elevated following Reperfusion

The baseline clinical characteristics of patients with MI are listed in Table 1. The mean age of patients with MI was 58.6 years, 88% were males, and the most prevalent pre-existing comorbidities were hypertension (46%) and hyperlipidemia (44%). Additionally, the baseline left ventricular ejection fraction was 52.1 ± 6.5% (Table 1). The maximal blood levels of cardiac enzymes (i.e., creatine kinase (CK), CKMB) and troponin in patients with MI are listed in the Supplementary Table S1.

**Figure 2:**
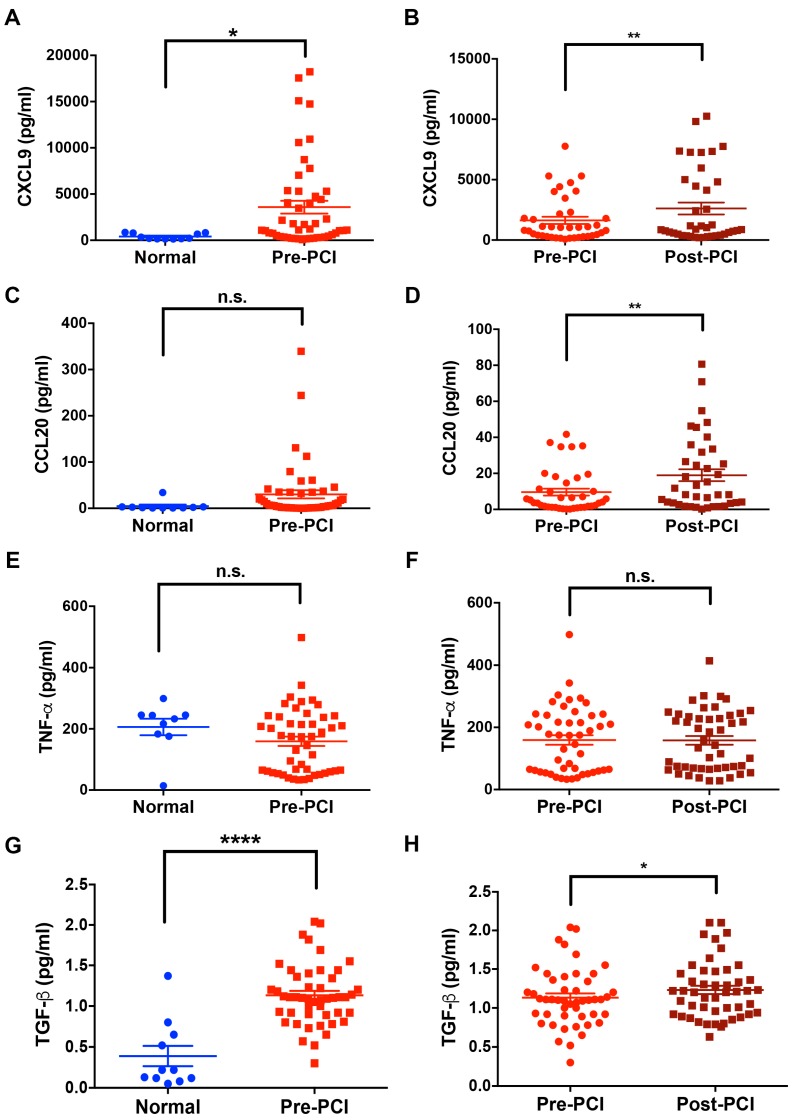
Expression of CXCL9 and CCL20 before (pre-PCI) and 3 h after coronary reperfusion by PCI (post-PCI) in patients with MI (*n* = 39–50) compared to normal controls (healthy volunteers in our laboratory) (*n* = 11). CXCL9 and CCL20 were measured by ELISA. (**A**) Expression of CXCL9 in normal controls and patients with MI. (**B**) Serum CXCL9 levels post-PCI in patients with MI remained more elevated than pre-PCI. (**C**) Serum CCL20 levels in normal controls and patients with MI. (**D**) Serum CCL20 levels change significantly after PCI. (**E**) Serum TNF-α levels in normal controls and patients with MI. (**F**) Serum TNF-α levels did not change significantly after PCI; (**G**) Serum TGF-β levels in normal controls and patients with MI. (**H**) Serum TGF-β levels in post-PCI MI patients remained more elevated than pre-PCI. ELISA = enzyme-linked immunosorbent assay; MI = myocardial infarction; TGF-β = transforming growth factor-β; TNF-α = tumor necrosis factor-α; PCI = percutaneous coronary intervention. Results are presented as mean ± SEM.

The serum levels of CXCL9, CCL20, TNF-α, and TGF-β in patients with MI (*n* = 39–50) and healthy controls (*n* = 11) were determined by ELISA (Figure 2). Compared to healthy controls (410.8 ± 91.66 pg/mL), patients with MI had a significant increase of serum CXCL9 (3584 ± 695.1 pg/mL) (*p* < 0.05) (Figure 2A). Additionally, the post-PCI CXCL9 levels were higher than the pre-PCI CXCL9 levels (2614 ± 489.2 pg/mL and 1635 ± 297.1 pg/mL, respectively, *p* < 0.001) (Figure 2B). Though there was a trend toward increased serum CCL20 in patients with MI (30.23 ± 8.88 pg/mL) compared to healthy controls (4.98 ± 3.236 pg/mL) (Figure 2C), it did not reach statistical significance. The serum CCL20 levels of post-PCI status were significantly higher than that of pre-PCI status (18.99 ± 3.304 pg/mL and 9.63 ± 1.916 pg/mL, respectively, *p* < 0.001, Figure 2D). The serum levels of TNF-α were not significantly increased in patients with MI (Figure 2E, and 2F). In contrast to TNF-α, patients with MI had a significant increase of serum TGF-β (1.13 ± 0.05 pg/mL) compared to normal controls (0.39 ± 0.12 pg/mL) (*p* < 0.0001) (Figure 2G). Additionally, the post-PCI TGF-β levels were more elevated than the pre-PCI TGF-β levels (1.23 ± 0.053 pg/mL and 1.13 ± 0.054 pg/mL, respectively, *p* < 0.05) (Figure 2H).

### 3.3. TNF-α and TGF-β Enhanced the Expression of CXCL9 and CCL20 in PBMCs

**Figure 3:**
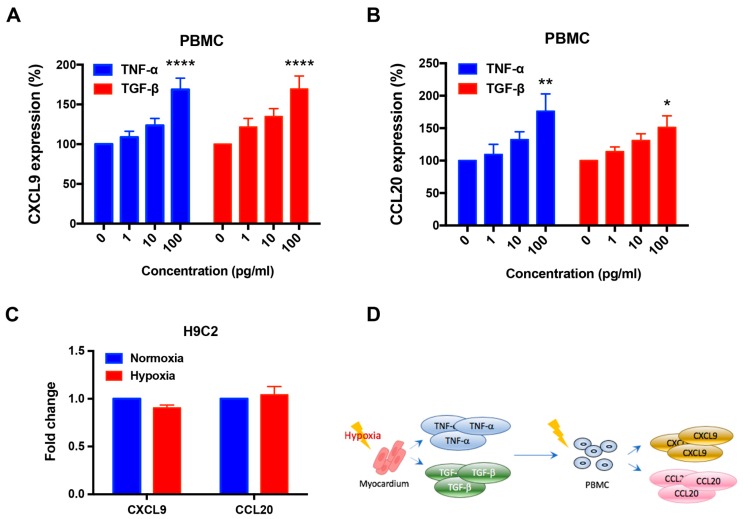
Expression of CXCL9 and CCL20 in PBMCs and H9c2 cells. (**A**) TNF-α and TGF-β upregulated the expression of CXCL9 in PBMCs. (**B**) TNF-α enhanced the expression of CCL20 in PBMCs. (**C**) H9c2 cells did not show an increased expression of CXCL9 and CCL20 under hypoxic stress. (**D**) Hypothetical illustration of the expression of CXCL9 and CCL20 after the onset of hypoxia. PBMCs = peripheral blood mononuclear cells; TGF-β = transforming growth factor-β; TNF-α = tumor necrosis factor-α. Each experiment was conducted at least 3 times independently. Results are presented as mean ± SEM.

Previous studies have demonstrated that CXCL9 and CCL20 were commonly secreted by PBMCs [25,26,27,28]. Here, we examined the expression of CXCL9 and CCL20 by PBMCs incubated with TNF-α or TGF-β (Figure 3). Recombinant TNF-α (100 pg/mL) significantly enhanced the expression of CXCL9 (1.6-fold increase, *p* < 0.0001) (Figure 3A) and CCL20 (1.76-fold increase, *p* < 0.01) (Figure 3B) by PBMCs. Recombinant TGF-β (100 pg/mL) also significantly enhanced the expression of CXCL9 (1.69-fold increase, *p* < 0.0001) (Figure 3A) and CCL20 (1.5-fold increase, *p* < 0.05) (Figure 3B).

### 3.4. The Expression of CXCL9 and CCL20 Was Not Increased by Hypoxia in H9c2 Cells

H9c2 cells incubated for 24 h under hypoxic conditions did not show an increase in the expression of CXCL9 and CCL20 compared to cells maintained in normoxia (Figure 3C). These findings indicated that CXCL9 and CCL20 were not primarily expressed by cardiomyocytes during an ischemic event. Taken together, our findings with PBMCs and H9c2 cells suggest that the enhanced levels of serum CXCL9 and CCL20 following MI were derived primarily from TNF-α- and TGF-β-activated PBMCs, not by cardiomyocytes (Figure 3D).

### 3.5. CXCL9 and CCL20 Enhanced the Growth and Migration of NHCFs

**Figure 4:**
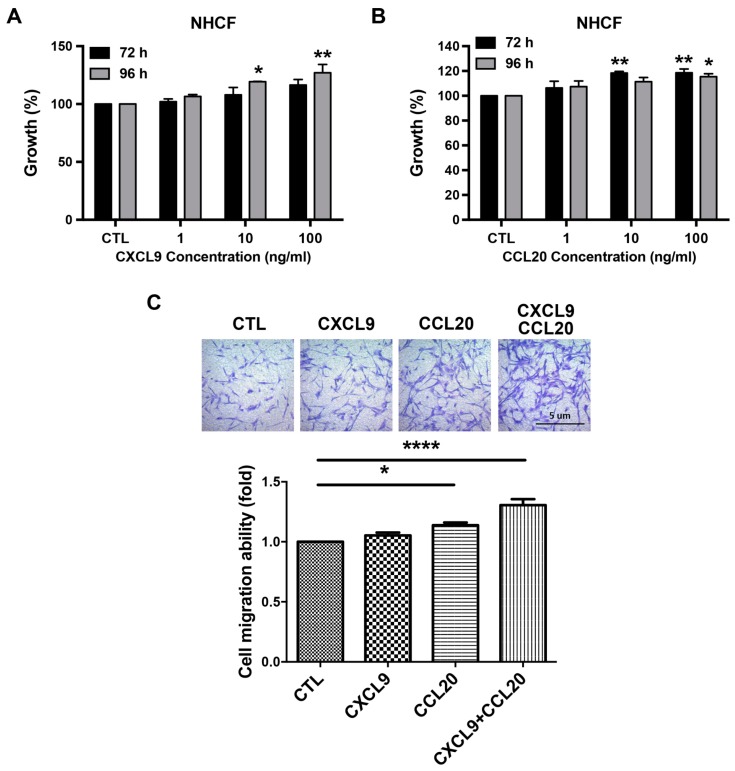
Effects of CXCL9 and CCL20 on the growth and migration of cardiac fibroblast. (**A**) A 96-hour incubation with CXCL9 (10 and 100 ng/mL) promoted the growth of NHCFs. (**B**) A 72-hour incubation with CCL20 (10 and 100 ng/mL), as well as a 96-hour incubation with CCL20 (100 ng/mL), promoted the growth of NHCFs. (**C**) CXCL9 and CCL20 promoted NHCFs migration. Detailed procedures are described in Materials and Methods. (Upper panel) Representative images of migrated NHCFs. (Lower panel) Quantitative results of the migration ability of NHCFs. CTL = control; NHCFs = Normal human cardiac fibroblasts. Each experiment was conducted at least 3 times independently. Results are presented as mean ± SEM.

To examine the effects of CXCL9 and CCL20 on fibroblasts, the growth of NHCFs incubated with recombinant CXCL9 and CCL20 was analyzed by the SRB assay. We observed that a 96-hour incubation with recombinant CXCL9 (Figure 4A) or recombinant CCL20 (Figure 4B) significantly enhanced the growth of NHCFs (CXCL9, 10 ng/mL: 119.34%, *p* < 0.05; CXCL9, 100 ng/mL: 127.05%, *p* < 0.01; CCL20, 100 ng/mL: 115.42%, *p* < 0.05). Additionally, we found that a 72-hour incubation with recombinant CCL20 significantly enhanced the growth of NHCFs (CCL20, 10 ng/mL: 111.33%, *p* < 0.01; CCL20, 100 ng/mL: 115.42%, *p* < 0.01) (Figure 4B). In the migration assay, 10 ng/mL CXCL9 and/or CCL20 significantly upregulated the migration ability of NHCFs (CXCL9, 10 ng/mL: 1.05 ± 0.02-fold increase, *p* < 0.05; CCL20, 10 ng/mL: 1.14 ± 0.02-fold increase, *p* < 0.05; combination of CXCL9 and CCL20, 10 ng/mL of each: 1.31 ± 0.05-fold increase, *p* < 0.0001) (Figure 4C).

### 3.6. Increased Serum CXCL9 and NT-ProBNP in Rats with ISO-Induced Cardiac Fibrosis

**Figure 5:**
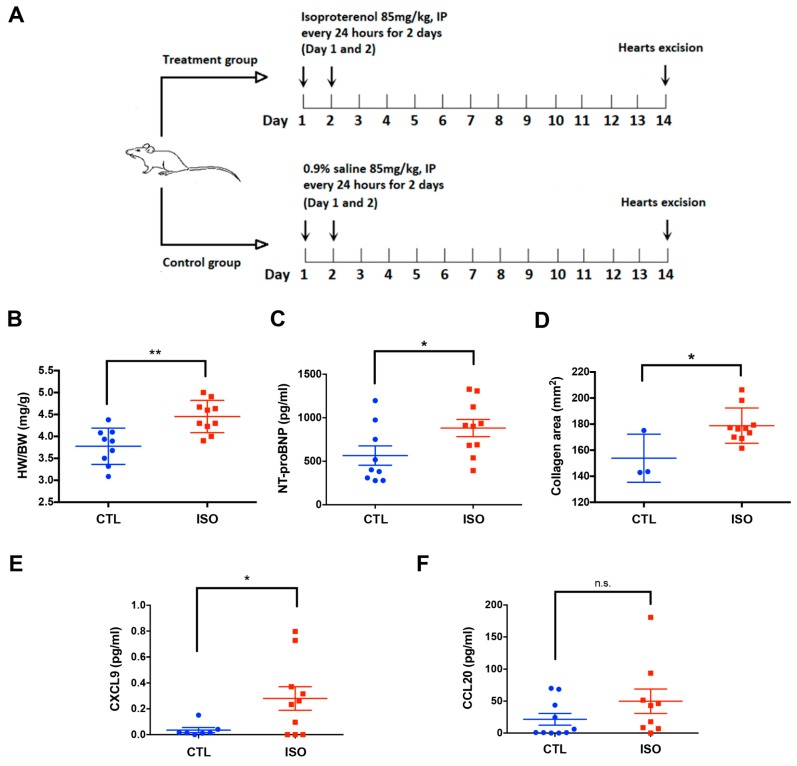
ISO-induced MI and post-MI cardiac fibrosis of rats. (**A**) Protocol of ISO-induced MI in rats. (**B**) HW/BW ratios in ISO-treated and control rats. (**C**) Serum NT-proBNP levels are increased in ISO-treated rats. (**D**) Heart sections of ISO-treated rats exhibited more collagen areas than control rats. (**E**) Serum CXCL9 levels are increased in ISO-treated rats. (**F**) Serum CCL20 levels in ISO-treated rats are not significantly increased compared to control rats. BW = body weight; CTL = control; HW = heart weight; IP = intraperitoneal; ISO = isoproterenol; MI = myocardial infarction; NT-proBNP = N-terminal prohormone of brain natriuretic peptide. Results are presented as mean ± SEM.

Figure 5A shows the protocol of ISO-induced cardiac fibrosis in rats. Compared to control rats (*n* = 10), ISO-treated rats (*n* = 10) had a higher heart-weight to body-weight ratio (4.454 ± 0.1162 vs. 3.775 ± 0.1379 mg/g, *p* < 0.01) (Figure 5B), a higher serum level of NT-proBNP (881.3 ± 98.67 vs. 565.2 ± 111.4 pg/mL, *p* < 0.05) (Figure 5C), and larger collagen areas in the myocardium (178.8 ± 4.283 vs. 153.8 ± 10.63 mm^2^, *p* < 0.05) (Figure 5D). The serum CXCL9 levels in ISO-treated rats were increased compared to control rats (0.2803 ± 0.09123 vs. 0.03543 ± 0.01984 pg/mL, *p* < 0.05) (Figure 5E). Though there was a trend toward increased serum CCL20 in ISO-treated rats, it did not show statistical significance compared to control rats (Figure 5F).

**Figure 6:**
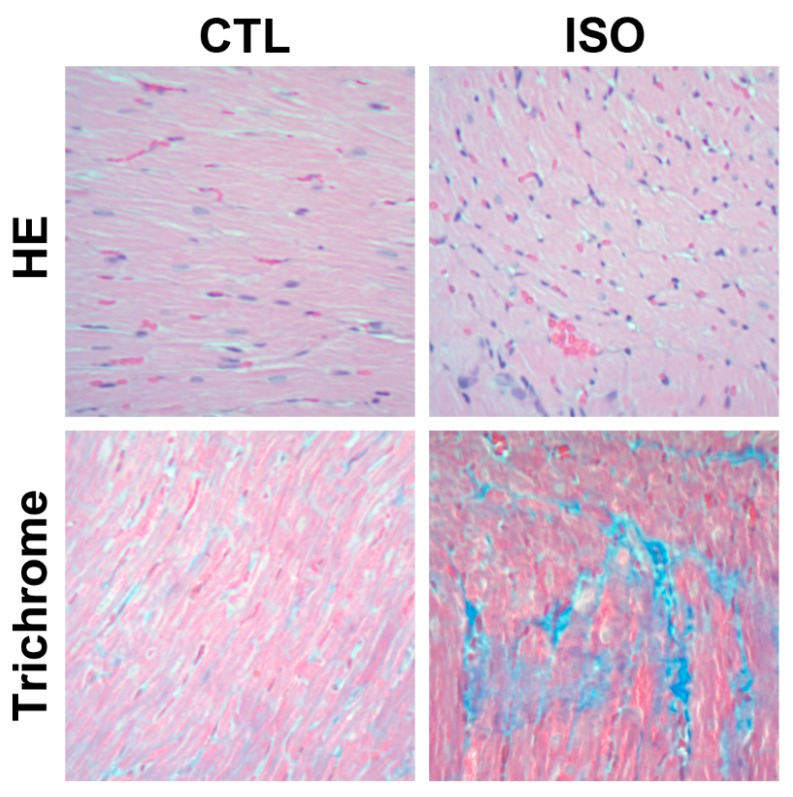
Representative HE- and trichrome-stained heart sections of rats under 200x magnification. CTL = control; HE = hematoxylin and eosin; ISO = isoproterenol.

HE-stained heart sections of ISO-treated rats showed diffuse irregular widening and disorganized arrangement of myofibrils compared to that of control rats (Figure 6). Additionally, trichrome-stained heart sections of ISO-treated rats showed marked collagen deposition (blue areas) compared to that of control rats (Figure 6).

## 4. Discussion

In the present study, we observed that patients with MI had significantly increased the serum levels of CXCL9 and TGF-β compared to normal individuals. Additionally, the serum CXCL9 and TGF-β levels in patients with MI remained upregulated after PCI. CXCL9 was vigorously expressed by TNF-α- and TGF-β-activated PBMCs but not by hypoxia-treated H9c2 cells. Rats with ISO-induced cardiac fibrosis had an increase of serum NT-proBNP and CXCL9 levels and a marked collagen deposition in the myocardium compared to control rats. Moreover, we found that CXCL9 enhanced the growth and migration of NHCFs in vitro. Collectively, the findings presented in this study indicated that an enhanced expression of CXCL9 following MI was involved in the pathogenesis of post-MI cardiac fibrosis.

Activated PBMCs are a major source of serum CXCL9 [25,26,27,28]. Pigs with simulated MI have a systemic activation of PBMCs [29]. In addition, PBMCs taken from patients with IHD produce a higher level of CXCL9 than PBMCs from normal subjects [14]. In the present study, TNF-α- and TGF-β-activated PBMCs expressed CXCL9 vigorously. Taken together, increased serum CXCL9 in patients with MI is suspected to be a specific downstream product of PBMCs that are activated by infarct-related cytokines.

Though TNF-α is a key cytokine that increases rapidly following MI [3,5,6], some investigators have reported that serum TNF-α levels do not change markedly within 12 h after admission in patients with MI undergoing reperfusion therapy [30,31]. This is consistent with the results of our current study. In contrast to TNF-α, we observed that the serum levels of TGF-β remained elevated in patients with MI after successful PCI. This finding suggested that activation of the TGF-β/PBMCs/CXCL9 axis might have a role in fibrotic remodeling of myocardium following MI, as demonstrated in several previous studies [11,30,32]. The benefits of complete inhibition of TGF-β signaling to ameliorate post-MI cardiac fibrosis remain controversial [32,33,34]. Ikeuchi and colleagues reported that inhibiting TGF-β in the late post-MI period attenuated ventricular remodeling and interstitial fibrosis, while inhibition in the early post-MI period exacerbated the degree of contractile dysfunction [33]. The results in the current study suggested that CXCL9 might be a more specific therapeutic target than TGF-β to prevent post-MI cardiac fibrosis.

In the present study, ISO-treated rats had pathological characteristics of post-MI cardiac fibrosis, as also shown by previous studies [22,23,24]. This animal model offers several advantages over the surgical model of coronary ligation, including simplicity, a low mortality rate, and a non-surgical technique thus eliminating the risk of procedure-related infections [22]. Oxidative stress, ischemia, intracellular calcium overload, metabolic changes, and alterations in the concentrations of electrolytes are considered as possible mechanisms of ISO-induced cardiac fibrosis [22,24]. We observed that patients with MI and ISO-treated rats with cardiac fibrosis had an increased level of serum CXCL9 compared to normal controls. These findings indicate that an increase of serum CXCL9 might be involved in the formation of cardiac fibrosis after infarction.

CXCL9 and its receptor, CXCR3, are associated with the progression of IHD and cardiomyopathy [13,14,15,16,17,18]. CXCL9 is highly expressed in atherosclerotic plaques of coronary arteries and specifically recruits CXCR3-bearing Th1 cells that increase the risk of plaque progression and the occurrences of MI [15,16]. A recent study of patients with idiopathic dilated cardiomyopathy showed that an increased serum CXCL9 was associated with worsening ventricular dysfunction [18]. Another study showed that the serum CXCL9 was increased in mice with pressure-overload cardiac hypertrophy, and in hypertensive patients with ventricular dysfunction [17]. Despite these findings from previous studies, the potential mechanisms of CXCL9-associated cardiac fibrosis remain unclear. The results presented in our current study provide valuable information that an increased level of serum CXCL9 following MI may activate proliferation and migration of cardiac fibroblasts, thus eventually leading to the formation of cardiac fibrosis. In addition to direct effects on cardiac fibroblasts, the recruitment of immune cells into the infarcted myocardium may possibly be involved in facilitating CXCL9-associated cardiac fibrosis [15,16,35,36,37]. Further investigations are needed to examine the interplay between the TGF-β/PBMCs/CXCL9 axis, cardiac fibroblasts, and immune cells in the pathogenesis of post-MI cardiac fibrosis.

Though some previous studies have shown that serum CCL20 levels are related to the severity of chronic inflammation and IHD [19,27,28], evidence of CCL20-associated cardiac fibrosis is very limited. In the present study, we observed that TGF-β-activated PBMCs had an enhanced expression of CCL20. Additionally, NHCFs treated with CCL20 exhibited an increase of proliferation and migration in vitro. However, the serum levels of CCL20 in patients with MI and rats with cardiac fibrosis were not significantly increased compared to normal controls. A potential reason for this finding might be the relatively small sample size used to reach statistical significance. Whether CCL20 is involved in the formation of post-MI cardiac fibrosis needs further research to be determined.

Our study is subjected to some limitations. First, the blood samples of patients with MI were only drawn before and 3 h after PCI. Therefore, we could not observe time-related changes of serum CXCL9 and CCL20 during the entire disease course of MI. Besides, we did not compare the serum expression of CXCL9/CCL20 and other well-known parameters (e.g., reperfusion quality and myocardial remodeling). Second, we did not perform endomyocardial biopsies in patients with MI to obtain histological information that could be correlated with the patients’ laboratory data. Third, we did not measure hemodynamic parameters in the animal experiments for comparison. In addition, the ISO-induced cardiac fibrosis model used in the present study does not fully reflect the actual clinical situations. Finally, we did not determine the blood levels of cardiac enzymes and troponin in animal experiments to validate the animal model used in the present study. Despite these limitations, the results of the present study provide valuable data that demonstrate the potential role of CXCL9 in modulating post-MI cardiac fibrosis.

## 5. Conclusions

Serum CXCL9 levels are enhanced following MI and remain elevated despite restoration of the coronary circulation. CXCL9 might be involved in the pathogenesis of post-MI cardiac fibrosis by activating the proliferation and migration of cardiac fibroblasts. These findings might be of clinical importance in identifying CXCL9 as a prognostic biomarker and a new therapeutic target to prevent post-MI cardiac fibrosis with ventricular dysfunction.

## Figures and Tables

**Table 1:** Baseline clinical characteristics of patients with MI receiving successful reperfusion treatment.

			Patients with MI *n* = 50
Age, Mean (± sd) (Years)			58.6 (± 10.4)
Male, n (%)			44 (88%)
**Clinical information, n (%)**			
	Current smoker		16 (32%)
	Diabetes		13 (26%)
	Hypertension		23 (46%)
	Hyperlipidemia		22 (44%)
**Prescribed medications before acute MI**			
	Aspirin		10 (20%)
	ACEI/ARB		6 (12%)
	CCB		4 (8%)
	Beta-blockers		2 (4%)
	Statin		3 (6%)
**Pre-PCI laboratory data, Mean (± sd)**			
	Cr (mg/dL)		1.18 (± 0.27)
	eGFR (mL/min)		69.2 (± 16.1)
	Total cholesterol (mg/dL)		193.1 (± 35.7)
	Triglyceride (mg/dL)		154.4 (± 63.4)
	LDL (mg/dL)		119.4 (± 27.4)
	HDL (mg/dL)		40.2 (± 9.39)
	Hb (g/dL)		14.1 (± 1.3)
	WBC count (/µL)		9629.7 (± 2630.5)
	Platelet (10^3^/µL)		245.9 (± 60.0)
	LVEF (%)		52.1 (± 6.5)
**Angiographic findings, n (%)**			
	Multi-vessels disease		17 (34%)
	Infarct-related artery		
		Left anterior descending artery	21 (42%)
		Left circumflex artery	9 (18%)
		Right coronary artery	20 (40%)

**Abbreviations:** ACEI/ARB = angiotensin-converting enzyme inhibitor/angiotensin II receptor blocker; CCB = calcium channel blocker; Cr = creatinine; eGFR = estimated glomerular filtration rate; LVEF = left ventricle ejection fraction; Hb = hemoglobin; HDL = high-density lipoprotein; LDL =l ow-density lipoprotein; min = minutes; LVEF = left ventricular ejection fraction; MI = myocardial infarction; PCI = percutaneous coronary intervention; sd = standard deviation; WBC = white blood cell.

## Supplementary Materials

The following are available online at https://www.mdpi.com/2077-0383/8/5/659/s1, Table S1: title, Maximal blood levels of CK, CKMB, and Troponin-I in patients with MI*.
