# Supplementary material for: Potential Effects of CXCL9 and CCL20 on Cardiac Fibrosis in Patients with Myocardial Infarction and Isoproterenol-Treated Rats
Source: J Clin Med. 2019 May 11;8(5):659. doi: 10.3390/jcm8050659 (PMC6572441; doi:10.3390/jcm8050659)
Supplement: Supplementary file 1 [file jcm-08-00659-s001.pdf]

Supplementary Materials

Supplementary Table S1. Maximal blood levels of CK, CKMB, and Troponin-I in patients with MI*.

No	Age	Gender	Presentation of MI	CK (U/L)	CKMB† (ng/mL)	Troponin-I‡ (ng/mL)
#1	74	F	NSTEMI	996	104.1	44.01
#2	51	M	STEMI	920	82.8	13.81
#3	88	M	STEMI	1155	15.1	1.52
#4	50	M	STEMI	3888	300	80
#5	55	M	STEMI	1032	157.6	29.46
#6	65	M	STEMI	1423	97.2	39.16
#7	60	F	STEMI	552	82.7	5.66
#8	38	M	STEMI	4604	298	76.47
#9	45	M	STEMI	1257	128.8	36.185
#10	44	M	STEMI	787	81.2	5.259
#11	58	M	STEMI	252	4.5	—
#12	49	M	STEMI	4510	300	—
#13	44	M	STEMI	3493	300	80
#14	56	M	STEMI	3675	300	6.291
#15	53	M	STEMI	1778	138.1	10.789
#16	72	F	STEMI	1695	118.8	27.314
#17	86	F	STEMI	2014	257.6	80
#18	55	M	STEMI	5491	300	80
#19	89	M	STEMI	1624	295	50.778
#20	51	M	STEMI	5421	300	42.927
#21	40	M	STEMI	3971	256.3	23.335
#22	77	M	STEMI	3080	168.9	0.344
#23	62	M	STEMI	1168	124.7	9.851
#24	58	M	STEMI	6299	300	80
#25	55	M	STEMI	3475	300	0.990
#26	68	M	STEMI	1730	168.4	1.000
#27	64	M	STEMI	1028	82.8	1.400
#28	55	M	STEMI	422	6.3	5.50
#29	49	M	NSTEMI	1404	166.4	1.800
#30	55	M	STEMI	106	16.2	0.912
#31	54	M	STEMI	894	104.3	25.629

#32	47	M	STEMI	1796	199	55.049
#33	50	M	STEMI	5684	300	80
#34	68	M	NSTEMI	309	37.0	0.980
#35	60	M	STEMI	11353	300	80
#36	56	M	STEMI	2029	151.4	11.307
#37	78	F	STEMI	254	32.1	4.442
#38	45	M	STEMI	1704	137.7	13.318
#39	69	M	NSTEMI	71	9.0	—
#40	41	M	NSTEMI	657	60.5	0.650
#41	83	M	STEMI	3240	300	29.466
#42	31	F	STEMI	2411	241.9	13.520
#43	50	M	STEMI	5256	300	80
#44	65	M	STEMI	2477	300	2.443
#45	62	M	NSTEMI	845	35	3.200
#46	62	M	NSTEMI	96	8.8	0.653
#47	70	M	STEMI	1049	104.8	9.750

Abbreviation: CK=creatine kinase; F=female; M=male; MI=myocardial infarction; N=Non-ST-elevation myocardial infarction; STEMI=ST-elevation myocardial infarction.

"—" indicated missing data.

*This table presented laboratory data of CK, CKMB, and Troponin-I from 47 patients with MI.

†CK value was presented as “300” when the patient’s blood CK levels were more than the upper limit value (300 ng/mL) in the laboratory of our institution.

‡Troponin-I value was presented as “80” when the patient’s blood Troponin-I levels were more than the upper limit value (80 ng/mL) in the laboratory of our institution.
